# Sex differences in the link between blood cobalt concentrations and insulin resistance in adults without diabetes

**DOI:** 10.1186/s12199-021-00966-w

**Published:** 2021-03-27

**Authors:** Yong Chen, Haobin Huang, Xiaowei He, Weiwei Duan, Xuming Mo

**Affiliations:** 1 Department of Cardiothoracic Surgery, Children’s Hospital of Nanjing Medical University, 72 Guangzhou Road, Nanjing, 210008 China; 2 Department of Cardiovascular Surgery, the First Affiliated Hospital, Nanjing Medical University, Nanjing, 210029 Jiangsu Province China; 3 Department of Endocrinology and Metabolism/Diabetes Care and Research Center, Nanjing Medical University Affiliated Geriatric Hospital/Jiangsu Province Geriatric Hospital, Jiangsu Province Official Hospital/Jiangsu Province Institute of Geriatrics, Nanjing, China; 4 Department of Bioinformatics, School of Biomedical Engineering and Informatics, Nanjing Medical University, 101 Longmian Avenue, Nanjing, 211166 China

**Keywords:** Cobalt, NHANES, Insulin resistance, HOMA-IR

## Abstract

**Background:**

Little is known about the effects of environmental cobalt exposure on insulin resistance (IR) in the general adult population. We investigated the association between cobalt concentration and IR.

**Methods:**

A total of 1281 subjects aged more than 20 years with complete blood cobalt data were identified from the National Health and Nutrition Examination Survey (NHANES) 2015–2016 cycle. Blood cobalt levels were analyzed for their association with IR among all populations and subgroups by sex. Regression coefficients and 95% confidence intervals (CIs) of blood cobalt concentrations in association with fasting glucose, insulin and homeostatic model assessment of insulin resistance (HOMA-IR) were estimated using multivariate linear regression after adjusting for age, sex, ethnicity, alcohol consumption, body mass index, education level, and household income. A multivariate generalized linear regression analysis was further carried out to explore the association between cobalt exposure and IR.

**Results:**

A negative association between blood cobalt concentration (coefficient = − 0.125, 95% CI − 0.234, − 0.015; *P* = 0.026) and HOMA-IR in female adults in the age- and sex-adjusted model was observed. However, no associations with HOMA-IR, fasting glucose, or insulin were found in the overall population. In the generalized linear models, participants with the lowest cobalt levels had a 2.74% (95% CI 0.04%, 5.50%) increase in HOMA-IR (*P* for trend = 0.031) compared with subjects with the highest cobalt levels. Restricted cubic spline regression suggested that a non-linear relationship may exist between blood cobalt and HOMA-IR.

**Conclusions:**

These results provide epidemiological evidence that low levels of blood cobalt are negatively associated with HOMA-IR in female adults.

**Supplementary Information:**

The online version contains supplementary material available at 10.1186/s12199-021-00966-w.

## Introduction

Insulin resistance (IR) is a condition in which normal concentrations of insulin cause a smaller than expected response in blood glucose levels [1]. Individuals with IR are susceptible to type 2 diabetes [2], and IR appears to play a crucial role in the pathogenesis of several diseases, such as polycystic ovary syndrome [3], Alzheimer’s disease [4], and cognitive dysfunction [5]. Although genetic predisposition [6], obesity [7], gut microbiota [8], and lifestyle [9] may evoke a disturbance in insulin sensitivity, various environmental factors also contribute to the risk of IR [10, 11]. Epidemiological studies have shown that exposure to several metals at chronic low levels was associated with a greater risk of IR [12]. However, the associations between blood cobalt (Co) and IR have not been studied.

Co is regarded as an essential trace mineral for all animals because it is the active center of a group of coenzymes, i.e., part of the B12 vitamin, which is important for human cell metabolism [13]. Generally, cobalt compounds are used as colorants in glass, ceramics, and paints; as catalysts; and as paint drying agents. Additionally, cobalt compounds are added into agricultural supplies and medicine as trace element additives. Cobalt can enter the body through the ingestion of contaminated food, respiration, skin absorption, and exposure to components of biomaterials [14]. However, it is only essential within a certain range, and a previous study has shown that normal serum values of cobalt are less than 0.5 μg/L [15]. However, these studies were experimental and examined only the short-term effects of excessive cobalt exposure.

In the present study, we explored the association between blood Co levels and IR using data from the National Health and Nutrition Examination Survey (NHANES), a nationwide survey of the general population in the USA. Moreover, subgroup analysis was performed to investigate sex differences.

## Methods

### Study subjects

The National Center for Health Statistics (NCHS; Centers for Disease Control and Prevention, Atlanta, GA, USA) conducted the NHANES studies. The NHANES protocols were approved by the NCHS Research Ethics Review Board, and a data user agreement was obtained from the website (https://www.cdc.gov/nchs/data_access/restrictions.htm). The NHANES is a cross-sectional study and contains a nationally representative sample of the non-institutionalized USA population. We selected only the data from the participants in the 2015–2016 NHANES for whom cobalt concentrations had been measured. The dataset included information on basic characteristics, a health questionnaire, laboratory data (i.e., blood cobalt, fasting glucose, and fasting insulin) and body measurements. All data used were retrieved from the website of the NCHS.

Populations in the NHANES over a range of 20 years were chosen to form a random subgroup for the detection of blood cobalt levels and IR. The two primary exclusion criteria included a missing fasting glucose or insulin measurement or an age less than 20 years. We also excluded women who were pregnant because they may have an abnormal physiological status that prevents accurate detection of IR. In addition, we excluded diabetic subjects because the diabetic condition would influence the IR status. The criteria for judging type 2 diabetes mellitus is as follows: (a) a fasting blood glucose level of greater than or equal to 7.0 mmol/l (126 mg/dl); (b) a 2-h plasma glucose level equal or greater than 11.1 mmol/l (200 mg/dl); and (c) the self-reported use of diabetes, insulin or oral hypoglycemic agents, as well as the presence of diabetic retinopathy.

### Blood cobalt and IR measurement

The method for measuring blood cobalt concentrations is described in detail elsewhere [16]. IR was estimated by serum analysis. Fasting glucose [17] and serum insulin levels were measured according to standard procedures.

### Covariates

To reduce bias in our results, we adjusted a priori confounders in the regression analyses in the present study. The confounders were as follows: age, sex, ethnicity, body mass index (BMI), education level, alcohol use, and poverty income ratio (PIR). BMI was divided into three categories: < 25 kg/m^2^, 25–30 kg/m^2^, and > 30 kg/m^2^. Alcohol consumption was classified by determining whether alcoholic drinks had been consumed within the past year. The PIR was represented as household income by the poverty guidelines specific to the survey year, which was categorized as low (< 1) or high (≥ 1).

### Statistical methods

Continuous variables and categorical variables are expressed as means ± standard deviations and frequencies, respectively. Because the data were skewed in nature, we transformed the blood cobalt levels to logarithmic form (Supplemental Figure 1), and blood cobalt was regarded as quartiles in further analyses. Regression coefficients (Beta) and 95% confidence intervals (CIs) were presented to reflect the association between blood cobalt concentration and IR in the age- and sex-adjusted and fully adjusted multiple variable linear regression models. Homeostatic model assessment of insulin resistance ((HOMA-IR) = [(fasting insulin (μU/ml) * fasting glucose (mmol/l)/22.5]) [18] was used to reflect the IR status. Logistic regression analyses were performed to investigate the association between blood cobalt concentration and IR. The cutoff point of HOMA-IR was 4.78 (the population-specific 75th percentile of HOMA-IR), according to a previous study [19]. We performed multivariable linear models to explore the associations between interquartile ratio increases (IQ ratio = 75th/25th percentiles of cobalt levels) in blood cobalt and HOMA-IR. Furthermore, we used ordinal variables as integer values to conduct the statistical tests for linear trends. The magnitudes of the above associations are the average percent difference in HOMA-IR within each IQ ratio group, which was grouped as the subjects’ cobalt variables. The formula of magnitudes was [(IQ ratio^Beta) − 1] * 100. Restricted cubic spline regression models were performed to investigate the nonlinear relationship between blood Co and HOMA-IR. We used SPSS version 20.0 (SPSS, Inc., Chicago, IL) to analyze all data. All two-sided *P* values < 0.05 were considered to indicate statistical significance.

## Results

**Fig. 1:**
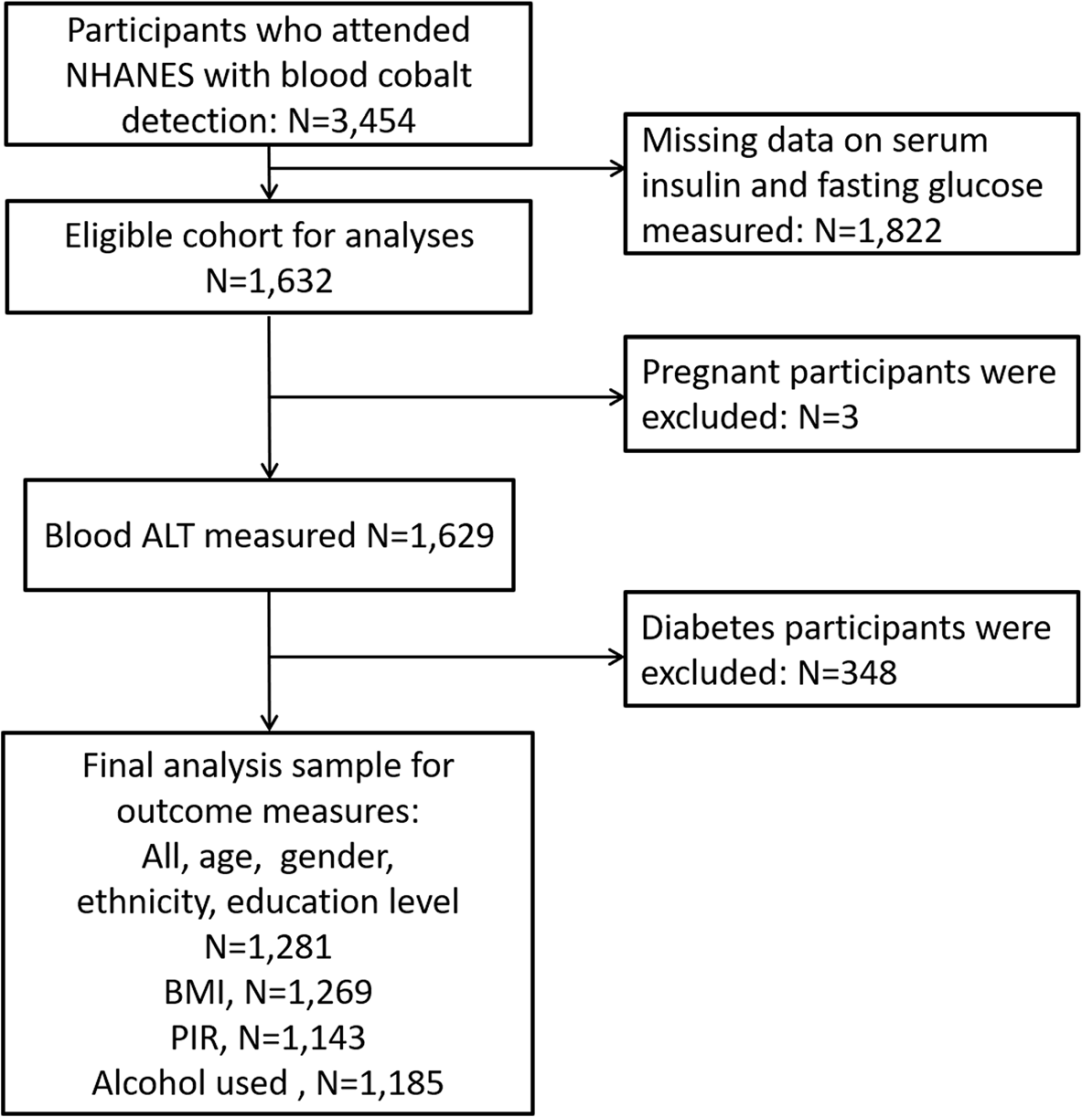
Eligible participants and those included in the analyses of the associations between blood cobalt exposure and insulin resistance in adults

The final investigation sample consisted of 1281 adults (720 males and 561 females) from this subgroup (Fig. 1). Table 1 shows that blood cobalt levels were significantly decreased among subjects who were less than 60 years old, were male, and consumed alcohol. The means ± standard deviations of HOMA-IR, fasting glucose, and insulin are listed in Supplemental Table 1 and classified by quartile of cobalt.

**Table 1:** Blood cobalt concentration (mean ± SD) according to demographics and lifestyle

	Cobalt (μg/L)		
	Participants [*n* (%)]	Mean ± SD	*P* value
Overall	1281 (100%)	0.20 ± 0.51	
Age (years)			< 0.001
< 60	720 (56.2%)	0.19 ± 0.57	
≥ 60	561 (43.8%)	0.21 ± 0.40	
Gender			< 0.001
Male	602 (47.0%)	0.18 ± 0.38	
Female	679 (53.0%)	0.22 ± 0.60	
Race			0.696
Mexican American	180 (14.1%)	0.26 ± 1.10	
Other Hispanic	180 (14.1%)	0.16 ± 0.13	
Non-Hispanic White	503 (39.3%)	0.20 ± 0.42	
Non-Hispanic Black	261 (20.4%)	0.17 ± 0.13	
Other race–including multiracial	157 (12.3%)	0.20 ± 0.27	
BMI (kg/m^2^)			0.061
< 25	345 (26.9%)	0.18 ± 0.30	
25–30	436 (34.0%)	0.23 ± 0.73	
> 30	488 (38.1%)	0.19 ± 0.35	
Alcohol use			0.001
Yes	800 (62.5%)	0.19 ± 0.34	
No	385 (30.1%)	0.22 ± 0.77	
PIR			0.248
< 1	233 (18.2%)	0.23 ± 0.97	
≥ 1	910 (71.0%)	0.19 ± 0.32	
Education level			0.228
Less than 9th grade	156 (12.2%)	0.27 ± 1.17	
9th–11th grade	146 (11.4%)	0.17 ± 0.25	
High school graduate/GED or equivalent	285 (22.2%)	0.20 ± 0.44	
Some college or AA degree	364 (28.4%)	0.19 ± 0.31	
College graduate or above	330 (25.8%)	0.18 ± 0.20	

The logistic regression results showed that cobalt concentration was not significantly associated with the risk of IR, regardless of whether the age- and sex-adjusted model or fully adjusted model was used (Supplemental Table 2). The linear regression results suggested that blood the cobalt concentration was negatively associated with the HOMA-IR index in females in age- and sex-adjusted models; however, the association moved substantially towards the null in the fully adjusted model (Table 2).

**Table 2:** Multivariable associations of blood cobalt concentrations with insulin resistance in US adults during 2015–2016

HOMA-IR		Model 1			Model 2		
		Coefficient	95% CI	*P* value	Coefficient	95% CI	*P* value
Continues variable	Overall	− 0.060	− 0.142, 0.023	0.157	− 0.063	− 0.137, 0.011	0.097
	Male	− 0.032	− 0.165, 0.100	0.631	− 0.082	− 0.199, 0.036	0.173
	Female	− 0.125	− 0.234, − 0.015	0.026	− 0.095	− 0.203, 0.012	0.082

Model 1: age and gender

Model 2: model 1 plus race, BMI, PIR, alcohol use, and education level

Exposure variables and risk factor variables were log transformed in the models

**Fig. 2:**
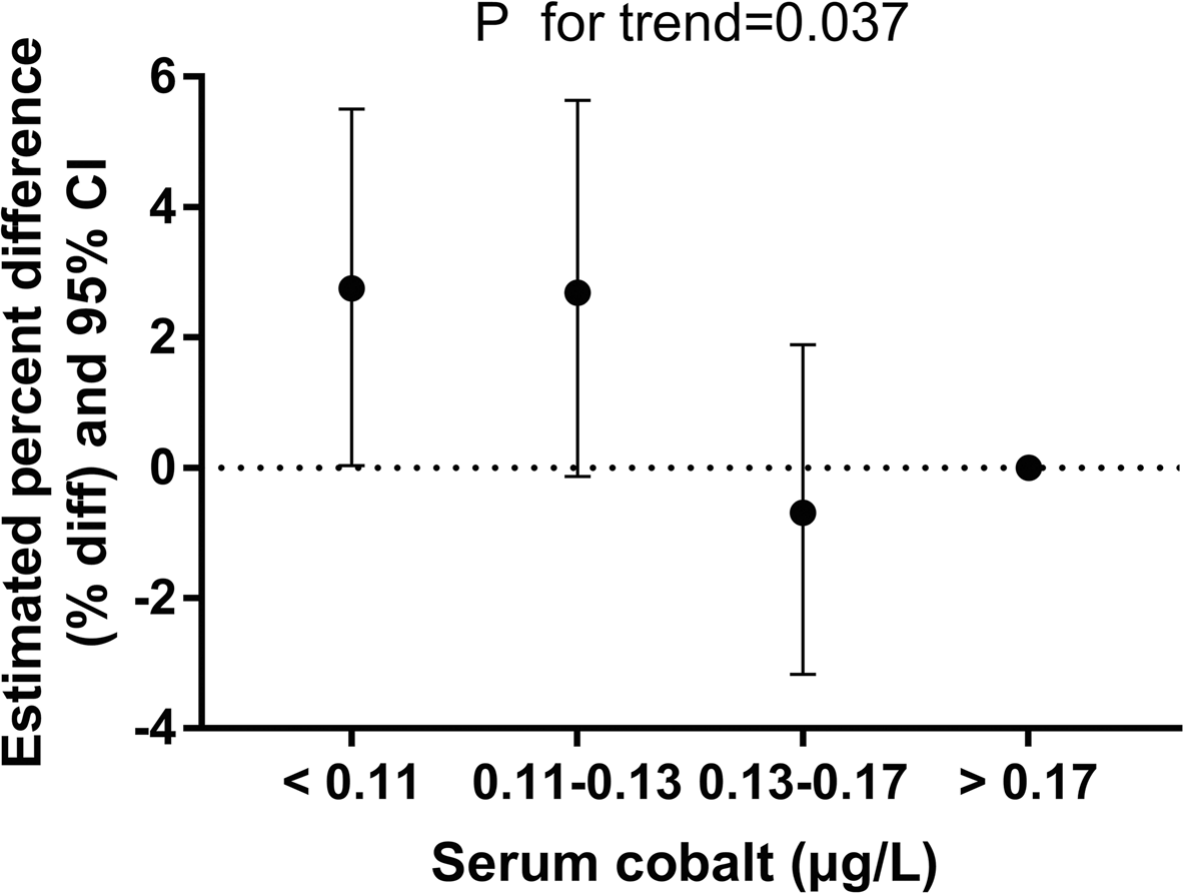
Estimated percent difference (% diff) and 95% confidence intervals (95% CI) in HOMA-IR in US adults during 2015–2016 for each interquartile ratio (IQ ratio) increase in blood cobalt levels

**Fig. 3:**
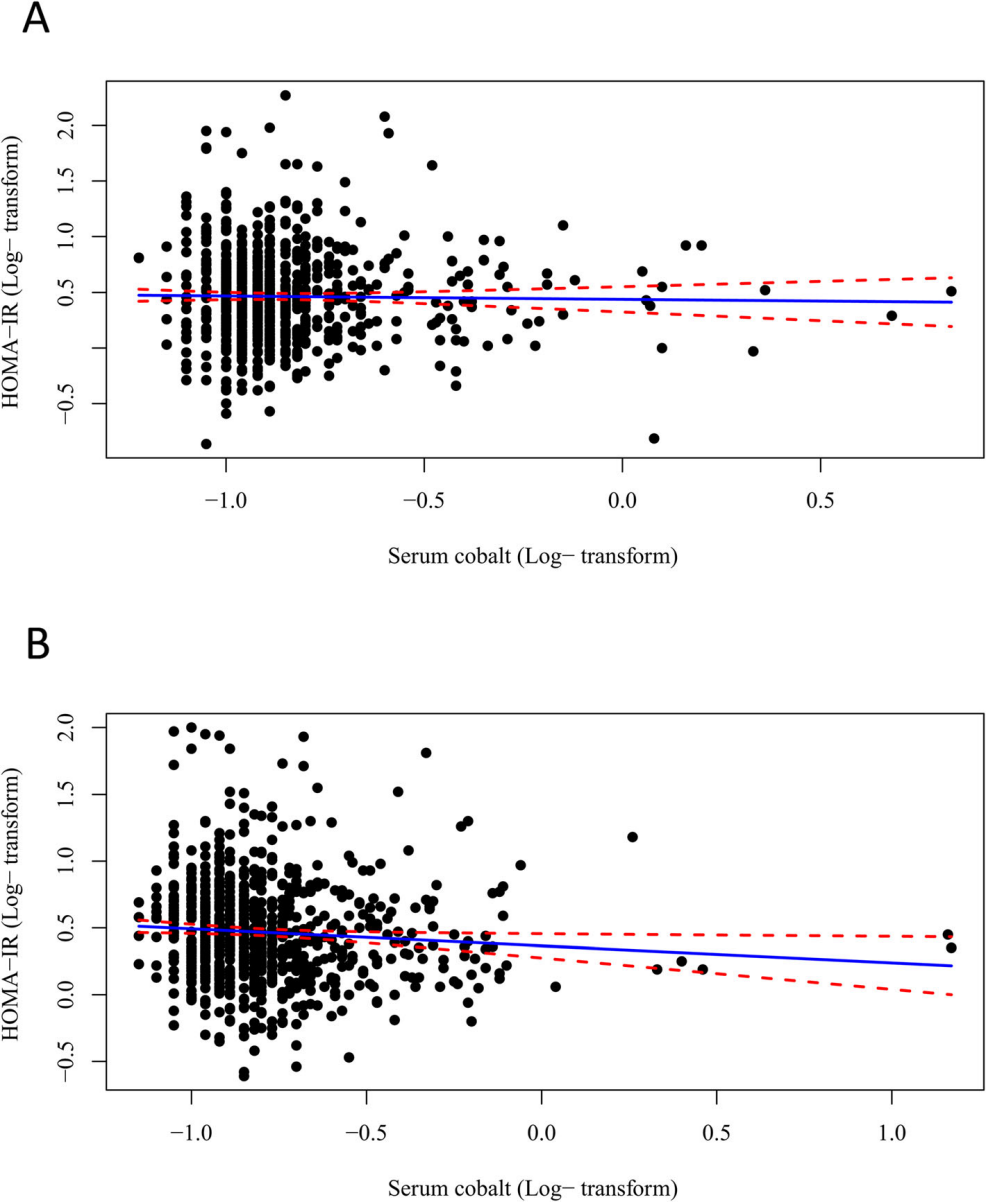
A scatter plot and a fitted line with 95% CI showing the relationship between blood cobalt levels and HOMA-IR in male and female adults

**Fig. 4:**
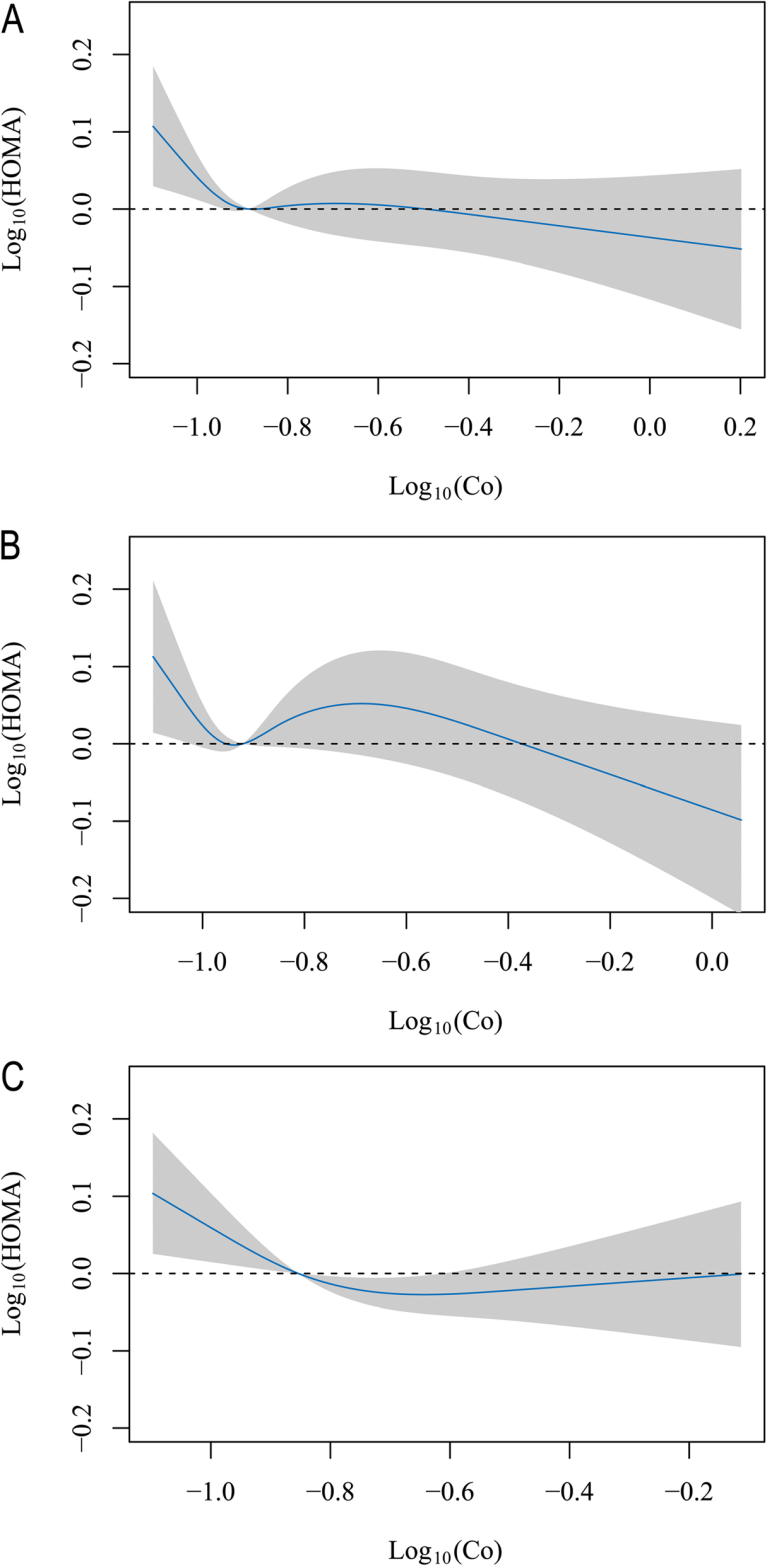
Predicted spline curves for the associations of HOMA-IR with blood Co concentrations according to restricted cubic spline regression models in the overall population (**a**), in males (**b**) and in females (**c**)

Table 3 indicates that blood cobalt concentrations in the lowest quartile compared with the highest quartile were positively associated with higher HOMA-IR (coefficient = 0.073, 95% CI 0.007, 0.139 for individuals under aged 60; coefficient = 0.062, 95% CI 0.001, 0.123 for females), with evidence of a dose-response relationship (*P* for trend = 0.016 and 0.037). Additionally, an association with blood cobalt concentration existed in the PIR ≥ 1 subgroup. Similarly, participants in the lowest cobalt quartile had a mean HOMA-IR that was 2.74% greater (95% CI 0.04%, 5.50%) than that in the highest quartile in females (Fig. 2). The relationship between blood cobalt and HOMA-IR in males and females is visualized by a scatter plot and fitted line with 95% CI (Fig. 3). Figure 4 shows the continuous relationship of blood Co with HOMA-IR based on the restricted cubic spline regression models. Significant nonlinear associations were detected between blood Co and HOMA-IR among males (*P* = 0.037) and females (*P* = 0.023), although the overall population did not seem to show a significant difference.

**Table 3:** Estimated coefficient (beta) and 95% confidence intervals (95% CI) of HOMA-IR in US adults during 2015–2016 for each quartile increase in blood cobalt levels stratified by different covariates

HOMA-IR	Quartile 1	Quartile 2	Quartile 3	Quartile 4	*P* for trend
All	0.038 (− 0.009, 0.084)	0.045 (− 0.004, 0.094)	0.03 (− 0.036, 0.061)	Reference	0.095
Age (years)					
< 60	**0.073 (0.007, 0.139)**	0.056 (− 0.013, 0.125)	0.022 (− 0.048, 0.093)	Reference	0.016
≥ 60	− 0.017 (− 0.083, 0.049)	0.030 (− 0.041, 0.101)	− 0.006 (− 0.073, 0.061)	Reference	0.696
Gender					
Male	0.020 (− 0.056, 0.096)	0.032 (− 0.049, 0.113)	0.061 (− 0.024, 0.146)	Reference	0.732
Female	**0.062 (0.001, 0.123)**	0.061 (− 0.003, 0.126)	− 0.016 (− 0.074, 0.043)	Reference	0.037
Ethnicity					
Mexican American	0.047 (− 0.063, 0.158)	− 0.026 (− 0.143, 0.091)	− 0.030 (− 0.145, 0.086)	Reference	0.779
Other Hispanic	0.062 (− 0.064, 0.188)	0.082 (− 0.040, 0.204)	0.057 (− 0.071, 0.185)	Reference	0.853
Non-Hispanic White	0.017 (− 0.053, 0.087)	0.050 (− 0.026, 0.125)	− 0.005 (− 0.074, 0.065)	Reference	0.279
Non-Hispanic Black	0.071 (− 0.050, 0.192)	0.083 (− 0.045, 0.211)	0.055 (− 0.087, 0.198)	Reference	0.146
Other race—including multiracial	0.049 (− 0.089, 0.187)	0.040 (− 0.110, 0.191)	0.021 (− 0.119, 0.160)	Reference	0.372
PIR					
< 1	− 0.035 (− 0.145, 0.075)	− 0.004 (− 0.119, 0.112)	− 0.023 (− 0.127, 0.082)	Reference	0.452
≥ 1	**0.055 (0.004, 0.106)**	0.057 (0.003, 0.112)	0.020 (− 0.035, 0.074)	Reference	0.015
Alcohol use					
Yes	0.032 (− 0.024, 0.089)	0.064 (0.003, 0.124)	0.038 (− 0.023, 0.099)	Reference	0.094
No	0.076 (− 0.007, 0.158)	0.016 (− 0.068, 0.101)	− 0.019 (− 0.099, 0.061)	Reference	0.382
BMI (kg/m^2^)					
< 25	0.090 (− 0.003, 0.183)	0.080 (− 0.018, 0.178)	0.097 (0.008, 0.186)	Reference	0.175
25–30	0.014 (− 0.058, 0.085)	0.063 (− 0.010, 0.135)	0.021 (− 0.053, 0.095)	Reference	0.177
> 30	0.029 (− 0.049, 0.107)	0.011 (− 0.075, 0.097)	− 0.059 (− 0.145, 0.028)	Reference	0.610
Education level					
Less than 9th grade	0.127 (− 0.004, 0.257)	0.041 (− 0.093, 0.174)	0.056 (− 0.074, 0.186)	Reference	0.205
9th–11th grade	0.102 (− 0.048, 0.252)	0.163 (− 0.001, 0.326)	0.129 (− 0.024, 0.283)	Reference	0.117
High school graduate/GED or equivalent	0.041 (− 0.065, 0.148)	0.013 (− 0.094, 0.120)	0.029 (− 0.076, 0.135)	Reference	0.710
Some college or AA degree	0.039 (− 0.046, 0.123)	0.073 (− 0.023, 0.168)	− 0.006 (− 0.097, 0.084)	Reference	0.386
College graduate or above	− 0.019 (− 0.099, 0.060)	0.017 (− 0.067, 0.101)	− 0.027 (− 0.116, 0.061)	Reference	0.718

Model was adjusted for age, gender, race, BMI, PIR, alcohol use, and education level.

*HOMA-IR* homeostatic model assessment of insulin resistance, *BMI* body mass index, *PIR* poverty index ratio

Cobalt (μg/L), quartile 1: < 0.11; quartile 2: 0.11–0.13; quartile 3: 0.13–0.17; quartile 4: > 0.17

## Discussion

We were the first to observe significant negative correlations between blood cobalt levels and HOMA-IR in the general female adult population.

Few studies have focused on the associations of cobalt with IR and type 2 diabetes. The review by Dubey et al. summarized existing research on cobalt concentrations in diabetic patients; however, there is not enough research currently, and the existing research has produced inconsistent conclusions [20]. Cao et al. found that elevated or decreased plasma cobalt levels were associated with a high risk of type 2 diabetes [21]. Anjum et al. found that blood cobalt concentrations in diabetic patients were higher than those in non-diabetic patients [22]. The urinary and serum concentrations of cobalt were decreased in individuals with type 2 diabetes compared with those in non-diabetic participants [23]. However, Menke et al. reported that higher quartiles of urinary cobalt were not associated with IR [12] and type 2 diabetes risk [24]. A positive association between cobalt and beta cell function was also observed, but it was not statistically significant [25]. Our findings from a relatively large sample suggest that cobalt may play a potential role in the IR process.

Excess cobalt exposure, such as during the treatment of cell lines or mice with excessive cobalt chloride, may act as a hypoxia-mimetic agent that can inhibit adiponectin transcription, thus contributing to the development of IR in vitro [26] and in vivo [27]. Symptoms of cobalt deficiency include hypoxia, growth retardation, weight loss, hepatic steatosis, anemia, immune dysfunction, reproductive dysfunction, and even death [28]. Low-dose cobalt, as a trace element, has been shown to assemble into enzymes such as cobalt protoporphyrin and attenuate IR [29] and improve insulin sensitivity [30, 31] in mice. The median blood cobalt concentration was lower than that in a previous occupational population [32], suggesting that the general public is likely not exposed to the same type or amount of cobalt dust that caused these effects in workers. In addition, 23 μg/L and 53 μg/L of cobalt in whole blood in men and women, respectively, did not cause alterations in hearing, vision, and cardiac and neurological functions [33]. Thus, an appropriate dose of cobalt in the subjects may have exerted beneficial effects in the present study.

Our results revealed negative associations between cobalt and HOMA-IR, and we thus speculated that proper cobalt intake may benefit insulin sensitivity. Cobalt-protoporphyrin IX treatment can improve endothelial function and insulin sensitivity by reducing oxidative stress, restoring the balance of eNOS/iNOS expression, and increasing the HO-1 level [34]. Another study showed that in the early stage of experimental diabetes, oral administration of 0.5 mM cobalt in drinking water reduced increases in the levels of thiobarbituric acid reactive substances (TBARS) and antioxidant enzyme activities in the heart and aorta [35]. In addition, we found that the associations were significant in female adults but not in male adults. Tvermoes et al. found that female adults had higher rates of cobalt absorption and lower rates of cobalt excretion than male adults [33], which is consistent with our findings that the cobalt levels in female adults were higher than those in male adults. We speculate that the reason for the sex difference in our study is the higher iron demand in women. A common intestinal uptake mechanism is used for cobalt and iron absorption. Animal and human studies have indicated that iron deficiency may increase cobalt absorption [36–39].

The present study has several critical strengths. While previous epidemiological studies of cobalt-related health effects were based on high exposure levels or small samples, the current study evaluated a relatively large sample with a non-occupational cobalt exposure level. In addition to the common covariates, potential factors that may generate bias in the results, for example, PIR, alcohol use and education level, were included in our study. Furthermore, we performed separate analyses stratified by sex to explore whether cobalt has effects on the sensitivity of different groups. Some limitations were unavoidable in the current investigation. Due to the nature of cross-sectional studies, we cannot distinguish whether blood cobalt influences IR or vice versa. Moreover, many environmental chemicals (such as phthalates [40], polycyclic aromatic hydrocarbons [41], polyfluoroalkyl chemicals [42], and bisphenol A [43]) are potentially associated with IR. These chemicals were not assessed in our analysis, which may have impacts on the association between cobalt and IR. Future studies are necessary to evaluate the interaction effect of different environmental chemicals on the risk of IR. Additionally, some information, such as cobalt contained in multivitamins and the presence of polycystic ovarian syndrome and type 1 diabetes, was not collected in the NHANES, so these factors could not be excluded in our study. These factors may affect the results and conclusions.

## Conclusions

In conclusion, the present study results indicated that blood cobalt exposure may be negatively associated with IR in the general US female adult population. Future research is needed to confirm this finding and investigate potential mechanisms.

## Supplementary Information

**Additional file 1.**

**Additional file 2: Table 1.** Insulin resistance indexes by quartile of cobalt concentration in US adults during 2015–2016.

**Additional file 3: Table 2.** Adjusted ORs (95% CIs) for the association between quartiles of blood cobalt concentration and HOMA-IR in all adults, stratified by gender.

## Data Availability

All data generated or analyzed during this study are included in this published article and its supplementary information files.

## Abbreviations

IR: Insulin resistance
CI: Confidence interval
HOMA-IR: Homeostatic model assessment of insulin resistance
NHANES: National Health and Nutrition Examination Survey
NCHS: National Center for Health Statistics
IQ: Interquartile
