# Supplementary figures and images for: Sex differences in the link between blood cobalt concentrations and insulin resistance in adults without diabetes
Source: Environ Health Prev Med. 2021 Mar 27;26:42. doi: 10.1186/s12199-021-00966-w (PMC8005238; doi:10.1186/s12199-021-00966-w)

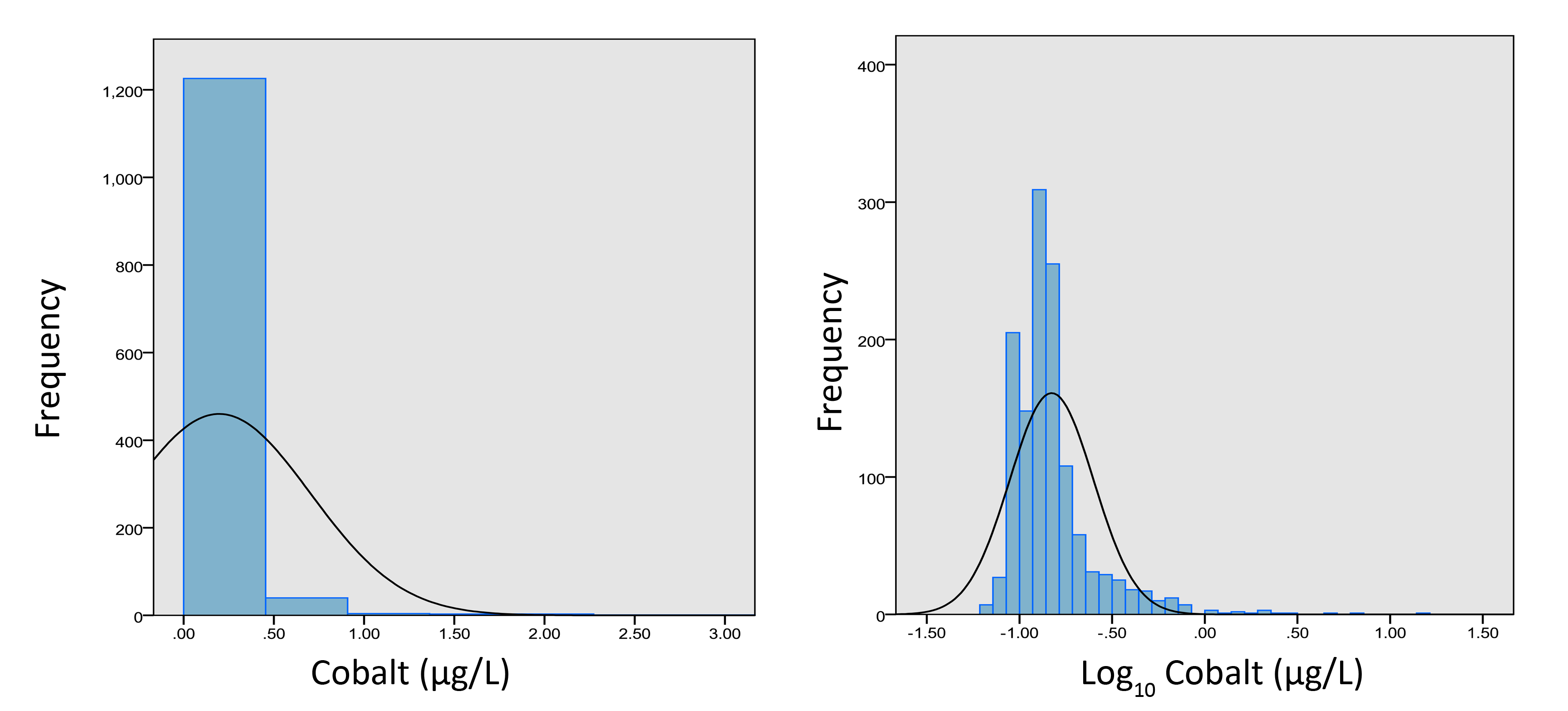

Supplement: Supplementary file 1 — Additional file 1. [file 12199_2021_966_MOESM1_ESM.tif]
