# Supplementary material for: Sex differences in the link between blood cobalt concentrations and insulin resistance in adults without diabetes
Source: Environ Health Prev Med. 2021 Mar 27;26:42. doi: 10.1186/s12199-021-00966-w (PMC8005238; doi:10.1186/s12199-021-00966-w)
Supplement: Supplementary file 2 — Additional file 2: Table 1. Insulin resistance indexes by quartile of cobalt concentration in US adults during 2015–2016. [file 12199_2021_966_MOESM2_ESM.doc]

**Supplemental Table 1.** Insulin resistance indexes by quartile of cobalt concentration in US adults during 2015–2016

| Characteristics | Cobalt | | | | |
| --- | --- | --- | --- | --- | --- |
|  | Quartile 1 | Quartile 2 | Quartile 3 | Quartile 4 | P value |
| HOMA-IR | 3.7±4.0 | 3.5±3.5 | 3.1±3.0 | 3.6±6.4 | 0.021 |
| Fasting glucose (mg/dL) | 106.3±15.3 | 107.8±23.5 | 107.5±27.4 | 105.9±23.4 | 0.028 |
| Insulin (µU/mL) | 13.3±11.9 | 12.4±9.9 | 11.2±9.2 | 12.9±17.3 | 0.025 |

Mean±SD

Cobalt (μg/L), quartile 1: <0.11; quartile 2: 0.11-0.13; quartile 3: 0.13-0.17; quartile 4: >0.17.
