# Supplementary material for: Sex differences in the link between blood cobalt concentrations and insulin resistance in adults without diabetes
Source: Environ Health Prev Med. 2021 Mar 27;26:42. doi: 10.1186/s12199-021-00966-w (PMC8005238; doi:10.1186/s12199-021-00966-w)
Supplement: Supplementary file 3 — Additional file 3: Table 2. Adjusted ORs (95% CIs) for the association between quartiles of blood cobalt concentration and HOMA-IR in all adults, stratified by gender. [file 12199_2021_966_MOESM3_ESM.docx]

**Supplemental Table 2.** Adjusted ORs (95% CIs) for the association between quartiles of blood cobalt concentration and HOMA-IR in all adults, stratified by gender

|  |  | Quartile 1 | Quartile 2 | Quartile 3 | Quartile 4 | P for trend |
| --- | --- | --- | --- | --- | --- | --- |
| All | Model 1 | 1.30 (0.95, 1.78) | 0.96 (0.68, 1.34) | 0.94 (0.67, 1.31) | Reference | 0.119 |
|  | Model 2 | 0.71 (0.48, 1.04) | 0.72 (0.49, 1.06) | 0.73 (0.49, 1.08) | Reference | 0.597 |
| Male | Model 1 | 1.13 (0.69, 1.83) | 0.73 (0.43, 1.24) | 0.96 (0.56, 1.64) | Reference | 0.633 |
|  | Model 2 | 0.60 (0.35, 1.03) | 1.06 (0.60, 1.87) | 0.69 (0.36, 1.31) | Reference | 0.285 |
| Female | Model 1 | 1.44 (0.94, 2.22) | 1.20 (0.76, 1.88) | 0.91 (0.59, 1.41) | Reference | 0.092 |
|  | Model 2 | 0.76 (0.43, 1.34) | 0.51 (0.29, 0.88) | 0.68 (0.40, 1.16) | Reference | 0.167 |

Cobalt (μg/L), quartile 1: <0.11; quartile 2: 0.11-0.13; quartile 3: 0.13-0.17; quartile 4: >0.17.

ORs were adjusted for age, gender, ethnicity, alcohol use, body mass index, education level, and household income.
